# Natural Killer Cells in Cancers of Respiratory System and Their Applications in Therapeutic Approaches

**DOI:** 10.1002/iid3.70079

**Published:** 2024-11-26

**Authors:** Maryam Dokhanchi, Atefe Panahipoor Javaherdehi, Mohammad Raad, Shayan Khalilollah, Pooya Mahdavi, Mohammad Hossein Razizadeh, Alireza Zafarani

**Affiliations:** ^1^ Department of Biology, Science and Research Branch Islamic Azad University Tehran Iran; ^2^ Department of Biotechnology, Tehran Medical Sciences Islamic Azad University Tehran Iran; ^3^ Department of Molecular, Cellular and Biomedical Sciences University of New Hampshire Durham New Hampshire USA; ^4^ School of Medicine, Tehran Medical Sciences Islamic Azad University Tehran Iran; ^5^ College of Public Health University of South Florida Tampa Florida USA; ^6^ Department of Virology, School of Medicine Iran University of Medical Sciences Tehran Iran; ^7^ Antimicrobial Resistance Research Center, Institute of Immunology and Infectious Diseases Iran University of Medical Sciences Tehran Iran; ^8^ Cellular and Molecular Research Center Iran University of Medical Sciences Tehran Iran; ^9^ Department of Hematology & Blood Banking, School of Allied Medicine Iran University of Medical Sciences Tehran Iran

**Keywords:** cancer, immunotherapy, natural killer cells, respiratory system

## Abstract

**Background:**

Cancer is still regarded as a major worldwide health issue due to its high health and socioeconomic burden. Currently, lung cancer is the most common cause of cancer‐related fatalities globally. Additionally, mesotheliomas and other cancers of the respiratory system, including those of the trachea, larynx, and bronchi, are also posing a significant health threat. Natural killer (NK) cells are lymphocytes of the innate immune system involved in response against cancer.

**Objective:**

This review discussed recent findings in the context of NK cell activity in the immune surveillance of respiratory system cancers and NK cell‐based treatments to combat those malignancies.

**Results:**

The presence of natural killer cells in the tumor microenvironment is shown to be associated with a higher survival rate in patients with various malignancies. However, cancerous cells benefit from several mechanisms to evade natural killer cell‐mediated cytotoxicity, including reduced major histocompatibility complex I expression, shedding of ligands, upregulation of inhibitory receptors, and release of soluble factors. Using NK cells to design therapeutic approaches may enhance antitumor immunity and improve clinical outcomes. Clinical trials investigating the use of natural killer cells in combination with cytokine stimulation or immune checkpoint inhibitors have exhibited promising results in various respiratory system malignancies.

**Conclusion:**

Respiratory system cancers present significant health challenges worldwide, and while NK cells play a crucial role in tumor surveillance, tumors often evade NK cell responses through various mechanisms. Advances in NK cell‐based therapies, including CAR‐NK cells, immune checkpoint inhibitors, and cytokine stimulation, have shown promising outcomes in tackling these tactics. However, challenges such as the immunosuppressive tumor microenvironment persist. Ongoing research is crucial to improve NK cell therapies by targeting autophagy, modulating miRNAs, and developing combinatorial approaches to enhance treatment efficacy for respiratory cancers.

## Graphical abstract

This article is a novel and comprehensive review of the roles of natural killer cells in various cancers of the respiratory system. It also discusses how those cells can be used to design cancer therapies to treat cancers of the respiratory system.

## 1 Introduction

Cancer is a major global health problem that poses a high socioeconomic burden on both developed and underdeveloped countries. Although the overall mortality is higher in opulent countries, there is a rising trend in cancer‐related mortality in underdeveloped nations [1]. The complexity of cancer highlights the importance of deciphering its mechanisms to pave the way for developing novel therapeutic approaches. The predominant respiratory system cancer is lung cancer, which is the leading cause of cancer‐related fatalities around the world by causing an estimated number of 2.2 million cases and 1.8 million deaths in 2020 [2]. It is associated with factors such as tobacco use and environmental exposure to hazardous materials. Cancers also affect other parts of the respiratory system including the trachea, larynx, and bronchi, with different incidence and survival outcomes [1].

Studying the antiviral and antitumor activity of the mouse immune system in the 1970s led to the discovery of natural killer (NK) cells. NK cells are one of the three types of major lymphoid cells in the blood [3]. In humans, NK cells stem from CD34^+^ progenitor cells in the bone marrow [4] and are characterized by expressing CD56 and lack of CD3 (CD3^−^ CD56^+^) [5]. This CD56 expression can be used to distinguish human NK cells from mouse NK cells, which do not express CD56 [6]. Also, the lack of CD3 and T cell receptor (TCR) distinguishes NK cells from T and NKT cells [4]. Furthermore, human NK cells are classified into two main groups: CD56^dim^ CD16^high^, which are highly cytotoxic cells that primarily dwell in the peripheral blood, and CD56^high^ CD16^dim^, which are located mainly in secondary lymphoid organs and are responsible for producing cytokines. NK cells are important parts of antiviral and anticancer immunity, and it is well‐established that their abundance in the tumor microenvironment (TME) is associated with more favorable survival in individuals with various malignancies [5].

While engineered T‐cell immunotherapies showed promising results in studies, their applications remain restricted to certain patients. Therefore, exploring other options, particularly innate immunity, has become an attractive option for researchers to explore [4]. Not being functionally restricted to the major histocompatibility complex (MHC) and having a high cytotoxic activity, NK cells are a highly potent candidate for the development of immunotherapies to treat cancer [7]. In this article, we review the role of NK cells in respiratory system tumors and therapeutic approaches utilizing NK cells.

## 2 Methodology

For this study, we searched the electronic databases of PubMed, Scopus, and Google Scholar to find relevant studies published up to August 2024, with the following search terms: (“Natural Killer cells” OR “NK cells”), AND (“respiratory cancer” OR “lung cancer” OR “tracheal cancer” OR “laryngeal cancer” OR “larynx cancer” OR “bronchial cancer” OR “pleomorphic sarcomas” OR “leiomyosarcomas” OR “mesothelioma”). We also did an additional search by searching through the reference lists of the included studies. After collecting the articles, articles in English languages that investigated NK cell activity in the context of cancers of respiratory system were included and articles without available full text or research on unrelated topics were excluded.

## 3 Respiratory System Cancers

There are many types of respiratory cancers, each of which poses challenges in diagnosis, treatment, and prognosis. Lung cancer is the most prevalent subtype and can be generally classified into two main categories: Nonsmall cell lung cancer (NSCLC) and small cell lung cancer (SCLC). NSCLC accounts for over 85% of all lung cancer cases [2]. Based on histological characteristics, NSCLC is further subdivided to different subclasses such as adenocarcinoma, squamous cell carcinoma (SCC), and large cell carcinoma [8]. Adenocarcinoma is often found in the lung periphery [9] and is more prevalent among women and nonsmokers [2]. SCC typically develops from the pseudostratified epithelium of proximal airways and is associated with a history of smoking, alcohol consumption, and infections that lead to chronic DNA damage such as human papillomavirus (HPV) and Epstein‐Barr virus (EBV) [10]. Large cell carcinoma is less common and is characterized by its poor prognosis, rapid growth, and tendency to metastasize early [11]. Large cell carcinoma mainly arises in the peripheral lung and is large (> 5 cm) and bulky [12]. Its development is highly associated with smoking [13]. SCLC is notorious for its aggressive nature and constitutes about 15% of lung cancer cases [14]. Unfortunately, SCLC is often diagnosed late at an advanced stage due to its rapid doubling time, which makes it hard to treat. SCLC is a neuroendocrine tumor and is strongly linked to smoking, with approximately 95% of cases occurring in individuals with a history of tobacco use [15]. Risk factors for respiratory system cancers are presented in Table 1.

**Table 1:** Risk factors for cancers of respiratory system.

Risk factor	Description	References
Tobacco smoke	The most well‐established risk factor for respiratory system cancers. It is linked to 80%–90% of lung cancer cases and contributes to other respiratory malignancies such as laryngeal and tracheal cancers.	[16, 17, 18, 19]
Environmental pollutants	Exposure to environmental pollutants, including air pollution and occupational hazards, significantly contributes to respiratory system cancers. Carcinogens like asbestos, radon, and industrial chemicals pose considerable risks.	[20, 21, 22]
Genetic factors	Genetic predisposition plays a crucial role. Mutations in tumor suppressor genes (e.g., TP53 in Li‐Fraumeni syndrome) and variants in DNA repair genes (e.g., BRCA1 and BRCA2) increase the susceptibility to respiratory malignancies.	[23, 24]
Chronic diseases and infections	COPD and chronic infections such as HPV are potential risk factors for respiratory cancers. COPD is a notable risk factor for lung cancer in nonsmokers, and HPV is linked to laryngeal cancer development.	[25, 26, 27]
Alcohol consumption	Alcohol consumption is recognized as a risk factor for laryngeal cancer.	[28]
Occupational exposures	Occupational exposure to carcinogens such as polycyclic aromatic hydrocarbons, diesel engine exhaust, organic solvents, and wood dust increases the risk of respiratory system cancers, particularly laryngeal cancer.	[29]

Abbreviations: COPD, chronic obstructive pulmonary disease; HPV, human papillomavirus.

There are other types of respiratory system cancers that arise beyond the lungs. One of them is tracheal cancer, which is a rare but distinctive malignancy occurring in the trachea. This cancer poses a diagnostic challenge by presenting nonspecific symptoms such as wheezing and dyspnea. Therefore, it is frequently diagnosed at advanced stages [30]. The majority (95%) of laryngeal cancer cases are SCC, which arises from the epithelial lining of the larynx, a crucial organ for airway protection and voice production [28]. Bronchial cancer is a diverse array of malignancies stemming from the bronchi. Its subtypes include bronchial adenomas, carcinoid tumors, and mucoepidermoid carcinomas, which exhibit distinct histological features [31, 32, 33, 34]. For instance, carcinoid tumors, which arise from neuroendocrine cells show a relatively indolent course [31]. The thoracic region is susceptible to sarcomas such as pleomorphic sarcomas and leiomyosarcomas, which are of clinical importance due to their scarcity and localization [35]. Additionally, mesotheliomas, which often happen in mesothelial cells lining the pleura, are rigidly associated with asbestos exposure [36].

## 4 Immunobiology of NK Cells

NK cells are innate lymphocytes that circulate in peripheral blood and also include several tissue‐resident phenotypes that have been identified in various parts of the body, including the lung [37, 38]. Also, Tissue‐resident NK cells were found in other different tissues such as the spleen, liver, and uterus with distinct lineages [37, 39]. Historically, NK cell classification relied on limited surface protein markers. However, a recent study employed advanced techniques, including single‐cell RNA sequencing (scRNA‐seq) and CITE‐seq, to further dissect NK cell heterogeneity. That study identified three prominent NK cell subsets in healthy human blood, labeled NK1, NK2, and NK3, which were divided into six subgroups based on molecular characteristics, key transcription factors, biological functions, and cytokine responses. These findings underscore that NK cells have diverse ontogenetic origins, leading to distinct transcriptional trajectories. Notably, the study also highlighted the distinct distribution of NK cell subsets in various tissues, including the lung, tonsils, and intraepithelial lymphocytes, as well as across 22 tumor types [40]. This structured classification system provides a foundation for future NK cell research to improve cross‐study comparisons.

NK cells can eliminate their targets through recognition of reduced MHC‐I levels, which is a signal that often indicates cellular distress or transformation, such as in virally infected or tumor cells. However, they can also use mechanisms that do not rely on MHC molecules, such as via activating receptors that recognize stress‐induced ligands on target cells. This dual capability allows NK cells to target a broad range of abnormal cells [41]. NK cells possess receptors that mediate their interactions with other cells. Notably, receptors such as killer cell immunoglobulin‐like receptors (KIRs), CD94/NKG2A, NKG2D, and NKp46 play crucial roles in cellular interactions between NK cells and nearby cells [42]. The “missing self” hypothesis suggests that these cells identify and remove cells with reduced or absent MHC I expression, which frequently happens in cells malignantly transformed or infected by viruses. This mechanism acts as a vigilant protector by preventing NK cells from mistakenly attacking healthy cells. Moreover, NK cells participate in “induced self” recognition by responding to the increased presence of stress‐induced ligands such as on the surface of target cells during cellular transformation or viral infection [43].

Aside from the mere receptor–ligand interactions, signals from inhibitory and activating receptors are integrated by NK cells to coordinate their functions. Inhibitory receptors, such as KIRs and CD94/NKG2iA, interact with self‐MHC molecules to avoid unwanted activation and response against healthy host cells [44]. On the other hand, activating receptors such as NKG2D identify stress‐induced ligands, viral proteins, or other changes on target cell surfaces to provoke NK cell activation [45]. There is a diverse array of inhibitory receptors such as NKG2A, Killer cell lectin‐like receptor subfamily G member 1 (KLRG1), KIRs, T cell immunoreceptor with Ig and ITIM domains (TIGIT), Human leukocyte antigens (HLA)‐G and lymphocyte activation gene 3 (LAG3) that abate NK cell cytotoxicity [46]. Recently, Nogo receptor 1 (NgR1), which is important in growth cone collapse, has been stated as a destructor of immunological synapses, and NgR1 deficiency or blockade has been shown to result in an improved NK cell to target cell contact stability and regulating F‐actin through immune synapse development, leading to enhanced antitumor effects of NK cells [47]. During stress response, the activation of the hypothalamic/pituitary adrenal axis results in the release of glucocorticoids in circulation. The presence of glucocorticoids in TME was shown to mitigate the antitumor function of NK cells by increasing programmed cell death protein 1 (PD‐1) expression [48]. Moreover, the presence of Galectin‐9 on NK cells was shown to be associated with higher cytotoxic molecules granzyme and perforin expression [49]. Noteworthy, cytokine signaling is another factor crucial in NK cell development. For example, interleukin (IL)−2 signaling through JAK1/3 and STAT pathways enhances NK cell proliferation and cytotoxicity [50], while IL‐15 promotes NK cell survival and activity [51]. Additionally, IL‐18 not only stimulates NK cell cytotoxicity and interferon (IFN)‐γ production but also protects NK cells from stress‐induced apoptosis [52].

Mice with B‐ and T‐cell deficiency showed the ability to mediate contact hypersensitivity responses against specific haptens in the second exposure [53]. This memory‐like feature has also been observed in the case of murine cytomegalovirus in mice and human cytomegalovirus, hantavirus, and chikungunya virus in humans [54, 55, 56, 57]. The development of memory‐like NK cells involves both receptor engagement and cytokine stimulation. A study reported that blocking inhibitory Ly49C/I during sensitization or challenge abrogated hapten‐mediated contact hypersensitivity in Rag1‐deficient mice. The study also found that antigens presented by MHC‐I, but not by MHC‐II, can elicit an NK‐mediated contact hypersensitivity response [58]. A study demonstrated that in human CMV (HCMV) infection, CD94/NKG2C^+^ NK cells expand and persist as memory cells. These NKG2C^+^ NK cells increase in number and also exhibit a more mature phenotype, characterized by the acquisition of CD57, a marker associated with memory and longevity in NK cells [59]. Moreover, there is evidence suggesting that activating KIRs, such as KIR2DS2 and KIR3DS1, may also play a role in the generation of memory NK cells, especially in individuals with the lack of NKG2C [60]. Cytokines were introduced as the main stimulants of memory‐like NK cells. Transferring cytokine‐activated murine NK cells to syngeneic mice showed enhanced interferon IFN‐γ production in comparison with not cytokine‐stimulated transferred NK cells [61]. Adaptively transferred murine cytokine‐induced memory‐like NK cells showed an improved response against tumors [62]. Noteworthy, contrary to conventional NK cells, the inhibition of memory‐like NK cells is KIR‐independent as blockade of KIRs showed no impact on the antitumor function of these cells. However, in vitro blockade of NKG2A enhanced antitumor response by memory‐like NK cells [63]. Circulating NK cells can be recruited upon skin infection with bacterial and viral pathogens and can develop into tissue‐resident memory‐like NK cells with faster response to secondary infections [64]. Memory‐like NK cells show some characteristics of adaptive immunity such as clonal expansion, robust antitumor response, longevity, and epigenetic modifications [65]. Therefore, they can be considered as promising options for cancer immunotherapies [66].

NK cells utilize various cytotoxicity mechanisms to destroy their target cells. A key mechanism is granule‐mediated cytotoxicity, wherein NK cells release perforin and granzymes upon activation. Perforin causes the formation of pores in the target cell membrane to allow the entrance of granzymes, which kill the target by inducing apoptosis. Simultaneously, NK cells use the death receptor pathway by expressing receptors such as Fas ligand (FasL) and tumor necrosis factor‐related apoptosis‐inducing ligand (TRAIL) on their surfaces. The interaction of these receptors with their respective ligands on target cells triggers apoptosis [67]. Furthermore, NK cells' FCγRIIIA (CD16) can bind to the Fc part of antibodies attached to a target cell and subsequently eliminate the target cell in a process called antibody‐dependent cellular cytotoxicity (ADCC) [68].

The aforementioned characteristics make NK cells interesting candidates in the development of cutting‐edge therapies. Techniques such as adoptive cell transfer involve expanding and activating NK cells ex vivo before reinfusing them into patients, leading to an increase in both the quantity and anticancer activity of NK cells, which often face inhibition in the TME. This approach has shown efficacy in various cancers, both hematologic malignancies and solid tumors [69]. Additionally, genetic engineering of NK cells, such as the expression of chimeric antigen receptors (CARs), is being explored to target specific cancer antigens. This can offer a tailored therapeutic strategy [70]. In addition, genetic engineering can be used to help NK cells penetrate the TME and avoid being inhibited by targeting chemokine receptors and inhibitory receptors [71]. Of note, as NK cells do not need to be specific to a particular patient, using them in immunotherapy offers an off‐the‐shelf method that makes them a powerful candidate to fight cancers [72]. Recently, NK cell‐derived exosomes have been introduced to the field of cancer immunotherapy given their safety and the easiness of modification [73]. Studies using NK cells to develop cancer therapies for respiratory system cancers are discussed in section 6.

## 5 NK Cells in Tumor Surveillance

The concept of cancer immunosurveillance was first presented almost half a century ago. It is widely believed that the immune system constantly monitors and controls instances of tumor cell transformation, which is seen as a frequent event. This hypothesis implies that those with inherited immunodeficiencies or those taking immunosuppressive therapies would have a significantly increased occurrence of cancer. Indeed, persons who have innate or acquired immunodeficiencies, as well as those who are taking immunosuppressive therapy, have a greater incidence of cancer [74]. The significance of abnormalities impacting T cells and innate immune system constituents has been highlighted in this context [75]. For example, patients with NK cell deficiency (NKD) are prone to developing cancer [76].

### 5.1 Anticancer Responses of NK Cells

Heightened cytotoxic activity and a higher count of peripheral NK cells are both correlated with a lower possibility of developing cancer [77]. This highlights the crucial role of the innate immune response in fighting against malignancies [78]. An essential function of NK cells is eliminating cells that display diminished or lacking expression of MHC. Within this particular framework, MHC class I molecules interact with inhibitory KIRs, which suppress the activity of NK cells, thereby preventing the excessive killing of healthy cells belonging to oneself. NK cells can detect and eliminate cancer cells more effectively due to the cancer cells' reduced expression of MHC [41]. Merely lacking MHC class I on the target cell is not adequate to initiate NK cell activation. Complete NK cell activation additionally necessitates the recognition of stress‐induced molecules by activating receptors present on NK cells [79]. Cancer cells can enhance the presentation of ligands that activate NK cell receptors. For example, the activation of NKG2D receptor ligands, such as MICA, MICB, and ULBPs, is mainly observed in cancer cells and during periods of cellular stress, infection, or DNA damage [80]. Furthermore, NK cells bind to cancerous cells through their NKp receptors and facilitate their destruction through lysis [81]. Another mechanism for target recognition and activation of NK cells involves the CD16 receptor, which binds to immunoglobulins' Fc region. When CD16 interacts with cells opsonized by immunoglobulins, it triggers a signaling cascade that ultimately destroys the antibody‐coated cell. This mechanism is a significant method by which NK cells eliminate cancerous cells [82]. Also, NK cells can activate other immune cells by secreting inflammatory cytokines to assist in killing cancerous or infected cells [83]. The impact of NK cells is not limited to their self‐actions against cancerous or infected cells. They can also collaborate with various other immune cells to invigorate the immune system. Through producing the chemokines CCL5, XCL1, and XCL2, NK cells enhance the trafficking of dendritic cells (DCs) into the TME [84]. NK cells play a role in developing DC within TME by producing FLT3LG, a crucial cytokine. The presence of FLT3LG and a high number of NK cells in tumors are associated with higher levels of DC, improved overall survival, and better responses to anti‐PD‐1 immunotherapy in patients with metastatic melanoma. Furthermore, there is a positive relationship between DC and NK cells, FLT3LG expression, T cell infiltration, increased survival, and the expression of checkpoint molecules. The production of FLT3LG by NK cells might help the survival of DC in the TME [5, 85]. Moreover, NK cells increase the antigen‐presenting capacity of DCs, which in turn enhances the activation and differentiation of T cells. T‐cell response is dependent on the number of antigens. In the presence of a low number of antigens, tolerance is induced while higher levels stimulate the response. By increasing and reducing the antigen presentation capacity of DCs, NK cells can respectively improve or hamper the cellular immunity response [86]. NK cells produce IFN‐γ, which leads to the enhancement of DCs antigen presentation and maturation and proliferation of B cells [5, 86]. Recently, a study examined NK cells in the TME across 716 cancer patients covering 24 tumor types by using single‐cell RNA sequencing analyses. This study revealed substantial heterogeneity in NK cell populations that are specific to the type of tumor. One key finding was the identification of a subset of tumor‐associated NK cells that exhibit impaired antitumor functions and are correlated with poor prognosis and resistance to immunotherapy. The study also identified that certain myeloid cell subpopulations, particularly LAMP3^+^ DCs, may regulate NK cell immunity against tumors [87]. This finding emphasizes on the complexity of relationship between NK cells and other immune cells within the TME, which may either hinder or enhance NK cell functionality.

### 5.2 Tumor Evasion of NK Cells Responses

Cancer cells employ evasion mechanisms by impeding the recruitment of NK cells into tumors. This is achieved through the presence of physical barriers created by tumor components, such as laminin and collagen [88]. Alternatively, cancer cells may selectively attract immature NK cells via a chemokine gradient [88]. A piece of research showed that the chemokine milieu in the TME is marked by reduced CXCL2, CX3CL1, CXCL1, and CXCL8, which attract CD56^dim^ NK cells. At the same time, there is an increased expression of CXCL9/10, CCL5, and CXCL19/21, which facilitate the movement of CD56^bright^ NK cells towards the stromal compartment [42]. Altered vascularization, leading to restricted food and oxygen supply, has been demonstrated to reduce the expression of activating NCRs. As a consequence, there is a decrease in the ability of NK cells to kill cancer cells and survive, hence weakening the antitumor actions of NK cells [89]. Tumors utilize numerous techniques to alter the normal functioning of NKG2D and avoid immune detection [90]. One method is the proteolytic shedding of the ligand, which results in the decrease or breakdown of NKG2D [91]. The released soluble form of MICA (sMICA) binds to NKG2D, resulting in its internalization. sMIC negatively impacts the expression of genes necessary for the balanced survival and development of NK cells [91]. Furthermore, ligands exist in exosomes secreted by cancer cells can reduce the expression of NKG2D [92]. TGF‐β and IDO1 were identified as factors that disrupt NK cell function by decreasing the expression of NKG2D in NK cells in TME [93, 94]. TME cytokines, such as TGF‐β, have the ability to potentially increase CD94/NKG2A expression [95]. Through NKG2A/CD94, IFN‐γ generated by tumors obstructs cytotoxic cells' ability to function [96]. The immune inhibitory effects of tumor IFN sensing operate through two key mechanisms. First, tumor‐induced upregulation of classical MHC class I inhibits NK cells. Second, IFN‐induced Qa‐1b expression suppresses CD8^+^ T cells via the NKG2A/CD94 receptor [96]. Furthermore, NKG2A expression is observed to be prevalent in NK cells located in the TME [97]. Certain tumor cells use elevated HLA‐E levels as a ruse to avoid the cytotoxic effects of NK cells [98]. The elevated expression of PD‐1 in NK cells is a common phenomenon observed in numerous cancer patients [99]. Within the TME, the PD‐L1 protein exhibits high expression levels. Notably, tumor cells strategically enhance local immune tolerance by overexpressing PD‐L1, a mechanism designed to regulate NK cells and T cells effectively [100]. PD‐L1 is prevalent in various cancer types and myeloid cells within the TME. For instance, a study has demonstrated that indirect suppression of NK cells occurs through PD‐L1/PD‐L2‐expressing TAMs, while direct inhibition is facilitated by malignant B cells [101]. Studies have shown the distinct function and importance of TIGIT in NK cells, emphasizing its increased expression in NK cells within the TME and its particular contribution to NK cell exhaustion [102, 103]. CD155, highly prevalent in numerous tumor tissues, functions through a complex mechanism in tumor immunity. When TIGIT binds to CD155, it can hinder the activities of NK cells by activating the PI3K and MAPK signaling pathways [104, 105]. In addition, microorganisms in the TME can impede the activity of NK cells by exploiting the TIGIT pathway. At a molecular level, TIGIT directly interacts with the fap2 protein of *Fusobacterium nucleatum*, a bacteria species often present in the TME, suppressing NK cell cytotoxicity [106]. Similarly, myeloid‐derived suppressor cells (MDSCs) found in the TME hinder the functioning of NK cells employing TIGIT [107]. A meta‐analysis of 53 studies revealed that the presence of NK cell infiltration is associated with a reduced risk of mortality in patients with solid tumors [108]. Among the four lung cancer papers analyzed, two studies indicated a notable association between high NK cell infiltration within the tumor and significantly enhanced overall survival. However, the remaining two studies did not observe any correlations between NK cell infiltration and prognosis [109, 110, 111, 112]. The disparities observed could be attributed to the TME hindering NK cell function through mechanisms beyond merely impeding their entry into the lung environment, as discussed previously. The findings underscore the complexity of the TME in lung cancers compared to other types of cancer and emphasize the pressing necessity to assess the TME in lung cancer cases.

## 6 Interplay Between NK Cells and Respiratory System Cancers

### 6.1 Lung Cancer

TME in the lungs comprises diverse elements such as tumor cells, cancer‐associated fibroblasts, and inhibitory immune cells such as Regulatory T cells (Treg), MDSCs, M2 macrophages, and N2 neutrophils. These inhibitory immune cells have been shown to hinder the efficacy of NK cells in the battle against lung cancer [113]. For example, MDSCs have the potential to hinder the activation of NK cells by inducing the proliferation of regulatory Treg cells through the activation of nitric oxide synthase (NOS), arginase, the generation of reactive oxygen species (ROS), and the reduction of L‐arginine, cysteine, and nitric oxide [114]. It has been demonstrated that Treg cells effectively suppress the antitumor activity of NK cells by hindering IL‐2‐mediated activation in NSCLC patients [115]. The vascular supply may be inefficient, leading to hypoxia and inadequate nutrient levels in the TME, which can negatively impact NK cell metabolism and their ability to exert cytotoxicity against tumors [116, 117].

**Figure 1:**
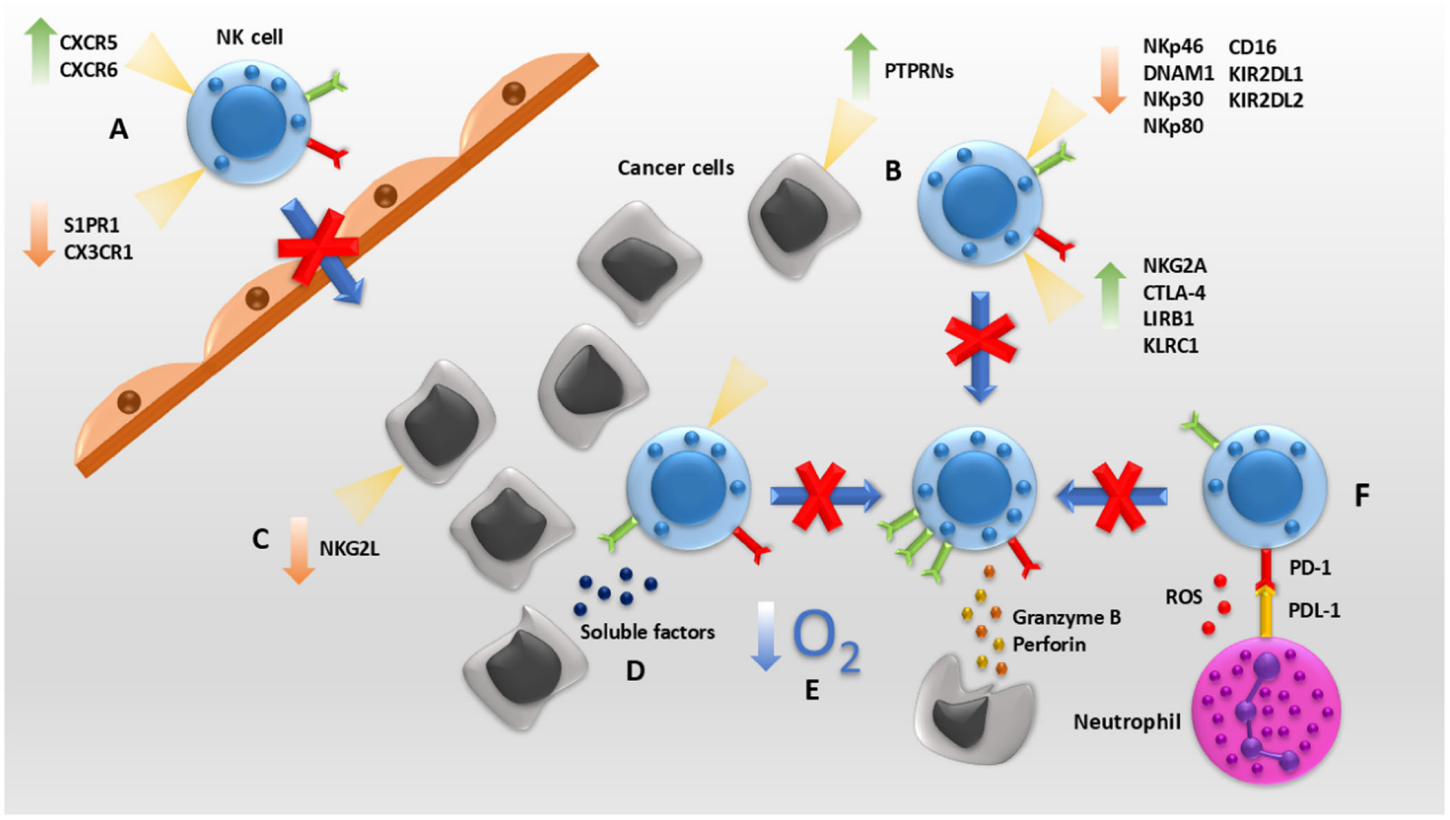
NK cell inhibition in respiratory system cancers happens through different ways: (A) Alterations in receptor expression impacts NK cell migration to the tumor microenvironment (TME), (B) Increasing NK cell inhibitory receptors and decreasing activating receptors expression by strategies such as expressing PTPRNs is used by cancer cells to avoid NK cell cytotoxicity. (C) Reduction of NKG2DL on cancer cell help these cells to evade being recognized by NK cells. (D) Cancer cells releasing soluble substances affecting antitumor activity of NK cells. (E) Hypoxia impedes the proper function of NK cells. (F) Other immune cells downregulate NK cell activity through binding to inhibitory receptors and releasing substances such as reactive oxygen species (ROS).

In individuals with lung cancer, a decrease in both the cytotoxic activity of NK cells and the production of cytokines has been observed. Additionally, the release of granzyme B by blood‐derived NK cells in lung cancer patients is diminished when compared to that of normal tissue [110, 118]. For example, Patients diagnosed with SCLC exhibited reduced cytotoxic activity in their NK cells, accompanied by a decrease in the expression of NKp46 and perforin, which might be related to the effect of TME on NK cells [118]. Research has demonstrated that the release of soluble substances by lung cancer cells can hinder the production of granzyme B, perforin, and IFN‐γ, and this interference is associated with the PGE2/COX2 pathway [119]. A summary of various ways of inhibiting NK cell activity in respiratory system cancers is shown in Figure 1. NSCLC patients exhibit reduced quantities of peripheral NK cells, characterized by a unique receptor expression pattern featuring decreased levels of NKp30, NKp80, CD16, DNAM1, KIR2DL1, and KIR2DL2, while NKp44, NKG2A, CD69, and HLA‐DR are upregulated. This altered receptor profile contributes to impaired cytotoxicity and facilitates tumor growth [120, 121]. Moreover, the progression of SCLC seems to be related to insufficient stimulatory signals to engage and activate NK cells, primarily because there is a reduction in NKG2DL. Primary SCLC tumors show minimal levels of NKG2DL mRNA, and cell lines derived from SCLC typically do not manifest NKG2DL expression at the protein level [122]. This process can be restored by the administration of a specific HDAC3 inhibitor and results in an elevation of NKG2DL expression on SCLC cells and enhances the cytotoxicity of NK cells [123]. A recent investigation indicated that a decrease in the expression of sphingosine‐1‐phosphate receptor 1 (S1PR1) and CX3CR1, accompanied by an increase CXCR5 and CXCR6, causes the impairment of NK cell immigration and increases CTLA‐4 and killer cell lectin‐like receptor (KLRC1) levels leads to the impairment of the NK cells' function within the TME of NSCLC [121]. Song et al. revealed an upregulation of receptor‐type tyrosine‐protein phosphatase‐like N (PTPRN) in NSCLC, showing a correlation with patient metastasis and a dampening effect on the cytotoxicity of NK cells. Elevated PTPRN levels enhance NSCLC cell migration and the expression of epithelial‐mesenchymal transition markers by influencing MEK/ERK and PI3K/AKT signaling pathways. This identifies PTPRN as a promising novel target for immunotherapy in NSCLC [124].

Tissue‐resident NK cells play a crucial role in the immune landscape of respiratory cancers, particularly in NSCLC. Recent studies have revealed that tissue‐resident NK cells accumulate significantly within the center of lung tumors, where they exhibit high granzyme expression and maintain functional responsiveness to target cell stimulation. Unlike CD8^+^ tissue‐resident memory T cells, tissue‐resident NK cells generally express lower levels of immune checkpoint receptors [125]. Additionally, a specific subset of tissue‐resident NK cells characterized by the expression of CD69 and CXCR6 has been identified within NSCLC tumors. Although this subset displays an immunomodulatory phenotype, it shows signs of functional exhaustion similar to T cells [126]. Moreover, a study on mice showed that tissue‐resident NK cells, including the predominant Mac‐1^hi^ CD27^lo^ subset, are instrumental in controlling both primary tumor growth and metastatic spread within the lungs. That shows their critical role in pulmonary tumor immunity [127]. Collectively, these studies suggest that tissue‐resident NK cells are key players in the defense against respiratory cancers and present promising options to be used in future cancer therapy studies.

### 6.2 Other Respiratory System Cancers

While lung cancer is the most studied respiratory malignancy due to its significant mortality and morbidity, NK cell activities were investigated in the context of other respiratory system malignancies including cancers of the larynx, bronchi, and mesothelium. It has been shown that the activity of NK cells has been reduced in patients with laryngeal cancer, which qualifies these cells for more investigation [128, 129]. For example, a study involving 60 patients with larynx and hypopharynx cancers showed that the activity of NK cells notably decreases in samples taken from blood and lymph nodes, particularly in the lymph nodes around the neck [130]. The decline in NK cell activity is linked to an unfavorable prognosis for patients with larynx cancer. Histological reports indicated that individuals who succumbed to the cancer exhibited lower NK cell activity compared to tumor‐free survivors [131]. Interestingly, a study revealed that there is no deficiency in NK cell numbers, as assessed by CD16 and CD56 markers, in patients with larynx cancer. However, consistent with other research findings, a reduction in NK cell activity was observed [132]. In a research study involving 51 cases of recurrent laryngeal cancer and 81 cases without recurrence, it was unexpectedly found that laryngeal cancer samples at high risk of recurrence showed increased infiltration of resting NK cells. Conversely, those at high risk of recurrence displayed decreased infiltration levels of activated NK cells [133].

The evaluation of PD‐L1 expression in primary tracheobronchial neoplasms revealed that not all types of these neoplasms exhibit an upregulation of PD‐L1. However, individuals diagnosed with SCC displayed an increased expression of PD‐L1 [134, 135]. Additionally, Zheng et al. demonstrated a higher density of FOXP3^+^ cells compared to cells expressing CD16 in the TME of SCC patients. This observation suggests a potential insufficiency or inadequate numbers of CD16‐expressing cells, including NK cells, in the TME [134].

Mesothelioma is the most studied type of outer lung respiratory system cancers. It has been shown that the survival outcomes in individuals with malignant pleural mesothelioma (MPM) did not exhibit notable variations based on the density of NK cells [109, 136]. For instance, a study identified abnormal infiltration of immune cells in patients with malignant MPM. Some patients exhibited only moderate infiltration of T cells or NK cells, and there was no correlation observed between the infiltration of immune cells and survival outcomes [136]. Interestingly, another study discovered a significant presence of infiltrated immune cells, including NK cells, in four instances of MPM [137]. Given that asbestos is a primary contributor to mesothelioma, understanding its impact on NK cell activity is crucial. Research indicates that exposing NK cells to asbestos leads to diminished cytotoxic activity, accompanied by changes in the expression of activating receptors such as NKG2D [138, 139]. Research on NK cells in patients with mesothelioma demonstrated a decline in cytotoxic activity and changes in receptor expression. Specifically, mesothelioma patients' NK cells displayed a distinctive reduction in NKp46 expression, while NKG2D and 2B4 exhibited normal expression, differing from the expression pattern observed in cell line NK cells [140]. Studies have indicated a correlation between alterations in NK cell cytotoxicity in mesothelioma patients and the phosphorylation of ERK1/2, which is recognized for triggering degranulation downstream of various NK cell‐activating receptors. Researchers demonstrated that individuals with elevated NK cell cytotoxicity exhibit a higher rate of ERK1/2 phosphorylation, whereas those with reduced NK cell cytotoxicity display a lower rate of ERK1/2 phosphorylation, which one of the effects of asbestos on NK cells is a reduction rate of ERK1/2 phosphorylation [138, 140].

In addition to respiratory system cells, immune cells such as NK cells, which are present in the respiratory tract, are prone to develop malignancies. The highly aggressive lymphoma known as extranodal NK cell lymphoma, nasal type (ENKL) is closely linked to the EBV and is most prevalent in Asia, South America, and Central America. In contrast, its incidence is low in the United States and Europe, comprising only 0.2%–0.4% of all newly diagnosed non‐Hodgkin lymphomas. During the diagnostic process, it is crucial to conduct polymerase chain reaction testing for EBV DNA in plasma and perform positron emission tomography/computer tomography and magnetic resonance imaging of the nasopharynx [141]. Individuals diagnosed with ENKL experience unfavorable outcomes. A study involving 123 patients with ENKL nasal type revealed that 73% achieved complete remission in stage I, while only 15% attained complete remission in stage IV, underscoring the aggressive nature of the disease [142]. Out of the 107 patients included in another study, 48 individuals (44.9%) successfully attained complete remission following the conclusion of their treatment. Over the course of the follow‐up, 60 patients (56.1%) succumbed to either local recurrence or metastases. The average survival duration was 70.0 months, the median survival duration was 42 months, and the 5‐year survival rate was recorded at 39.4% [143].

## 7 Therapeutic Implications

It has been recently shown that the activity or quantity of NK cells can serve as a valuable biomarker for predicting the response to immunotherapy in patients diagnosed with NSCLC and other cancers [144, 145, 146]. Research has demonstrated that the capacity of NK cells to produce inflammatory cytokines, particularly IFN‐γ, and express activating receptors such as NKp46, can serve as prognostic and protective indicators for immunotherapy [144, 145, 146]. After a thorough decade of investigation, it is now widely recognized that transferring activated NK cells improves the survival rates of individuals with lung cancer [147, 148]. For example, there is proof of prolonged survival in patients with NSCLC who underwent a combined adoptive transfer of T cells and NK cells that have been stimulated with cytokines [147]. Furthermore, individuals with advanced NSCLC have demonstrated enhanced functionality of immune cells and an improved quality of life after receiving ex vivo cytokine‐stimulated NK cells through allogeneic transfer [148]. The effectiveness and extended survival of patients with highly aggressive SCLC have been established through adoptive immunotherapy involving autologous NK cells, γ/δ T cells, and cytokine‐activated killer cells [149]. The utilization of IL‐15‐stimulated NK cells presents a promising allogeneic approach in the treatment of lung cancer patients [150]. For instance, the efficacy of NK cells activated by N‐803, an IL‐15 superagonist, has been demonstrated in effectively lysing SCLC tumor cells across various subtypes [151]. Additionally, the IL‐15 superagonist ALT‐803 has been shown to enhance NK cell growth, induce the expression of NKG2D, and stimulate the production and release of IFN‐γ. Combining ALT‐803 with the PD‐1 antibody nivolumab has suggested increased antitumor activity, progression‐free survival, and overall survival rather than using nivolumab alone [152, 153]. Recent findings indicate that tissue‐resident lung NK cells exhibit enhanced responsiveness to IL‐15, suggesting its potential as a viable option for patients with lung cancer or other malignancies [154]. In a randomized phase II clinical trial (EudraCT2008‐002130‐30), the efficacy of autologous ex vivo‐activated NK cells was assessed in individuals with NSCLC following radiochemotherapy. The findings indicate that autologous NK cells activated ex vivo with TKD/IL‐2 are well‐tolerated and yield favorable clinical outcomes in patients with advanced NSCLC post‐radiochemotherapy [155]. An important obstacle in adoptive NK cell immunotherapy is the requirement for a large number of NK cells. To overcome this challenge, other strategies have been developed, including the growth of autologous or allogeneic peripheral blood NK cells triggered by cytokines as discussed above. It is essential for adoptively transferred NK cells to maintain their capacity to eliminate tumor cells, while also effectively managing the activation and inhibition of receptor signaling. Additionally, it is important to closely monitor the occurrence of graft‐versus‐host disease (GvHD) in allogenic transfers. Therefore, it is necessary to carefully adjust various expansion techniques to guarantee the clinical efficacy of adoptively transferred NK cells [156, 157].

Immune checkpoint inhibitors have emerged as a valuable treatment choice for lung cancer. Recently, the therapy for individuals with NSCLC using inhibitors targeting immune checkpoint pathways, including PD‐1 (such as nivolumab) and PDL‐1 (including atezolizumab, durvalumab, avelumab), has shown enhanced clinical outcomes [158]. While the monoclonal antibody (mAb) targeting PD‐1 or PD‐L1 demonstrates effectiveness in treating NSCLC [159], not all patients with PD‐1/PD‐L1 positivity experience its benefits. The limited efficacy in some cases may be linked to compensatory upregulation of alternative immune checkpoints. The presence of inhibitory KIR in tumor cells is connected to PD‐1 expression. Therefore, a combined treatment involving both anti‐PD‐1/PD‐L1 and anti‐KIR could potentially offer assistance to patients who do not respond well to anti‐PD‐1/PD‐L1 therapy alone [160]. Notably, there have been investigations into the combined use of pembrolizumab (anti‐PD‐1) and NK cell transfusion. A total of 109 patients diagnosed with NSCLC were selected, with group A (55 patients receiving pembrolizumab along with NK cells) and group B (54 patients receiving pembrolizumab alone). The results indicate that the combination of pembrolizumab and NK cell therapy led to an enhanced survival rate in individuals with advanced NSCLC [161]. In SCLC patients, the administration of nivolumab and atezolizumab has been approved as first‐line treatment [162, 163]. Following the administration of tremelimumab, an anti‐CTLA‐4 treatment, the various types of NK cells (CD56^bright^ and CD56^dim^) in patients with mesothelioma reverted to normal physiological levels, as indicated by a study [164]. Nevertheless, these NK cell‐related immunotherapies are accompanied by certain drawbacks, including severe toxicity, limited efficacy in treating solid tumors, and inadequate long‐term durability [165]. Utilizing mAb treatment combined with ADCC stands out as a highly effective option for managing lung cancer patients. For example, in vitro experiments demonstrated that the combination of Avelumab and cetuximab (antiepidermal growth factor receptor) induced ADCC in NSCLC cells in the presence of NK cells [166]. An additional investigation involving an NSCLC cell line indicates that both NK cells and NK‐92 (an NK‐92 cell line transduced with CD16) can elicit an ADCC response when combined with anti‐PD‐L1 monoclonal antibodies. This combination enhances ADCC‐mediated antitumor activity, specifically against tumors expressing PD‐L1 [167]. A similar research study demonstrated that the use of irradiated NK‐92 cells, when adoptively transferred, enhances ADCC against lung carcinoma cell lines in combination with avelumab. The study also revealed that ADCC is impeded by the presence of an anti‐CD16 blocking antibody [168]. A promising collaboration between NK‐92 cells and mAb has been discovered in cell lines of NSCLC that are resistant to tyrosine kinase inhibitors (TKIs). The research demonstrated that the combined use of NK92 cells and cetuximab successfully eradicated TKI‐resistant NSCLC cells. These results suggest that a therapeutic approach combining NK cell‐based immunotherapy with cetuximab shows potential for individuals dealing with TKI‐resistant NSCLC [169]. MAb therapy is limited by various variables, including limited availability and accessibility, high costs, the need for early intervention, vulnerability to resistance and mutation, and the potential for side effects [170]. While clinical trials have shown promising response rates, a considerable number of NSCLC patients do not derive advantages from immune checkpoint inhibitor treatments. This lack of benefit may be attributed to factors such as an immunosuppressive microenvironment, insufficient activation of NK cells, a shortage of tumor‐specific NK cells, and a low mutational burden [171, 172, 173].

Tumors have the ability to avoid the activity of NK cells by decreasing the presence of ligands that activate receptors. They achieve this through different methods, including the release of exosomes, modifying glycosylation and lipidation patterns, enhancing intracellular retention, causing misfolding, and undergoing alternative splicing. In addition, malignancies have the ability to evade NK cell attacks by upregulating the production of MHC molecules, which interact with inhibitory receptors on NK cells [174, 175]. To address certain challenges, researchers have investigated the use of NK cells engineered with CARs to bolster NK cell activity through more robust signaling and targeted recognition. CARs represent a distinctive aspect of synthetic biology and bring about a transformative change in personalized medicine by leveraging the patients' immune cells to target and combat cancer. CAR‐NK cells combine the precise targeting of antigens with the subsequent intracellular signaling capabilities of receptors, enhancing their anticancer functionalities [176]. In comparison to CAR‐T cells, CAR‐NK cells present notable advantages. These include improved safety, with minimal or absent cytokine release syndrome and neurotoxicity in autologous scenarios, as well as a reduced risk of GvHD in allogeneic settings, these fewer side effects can be attributed to the shorter lifespan of NK cells and their production of fewer pro‐inflammatory cytokines [177]. Additionally, CAR‐NK cells offer multiple mechanisms for activating cytotoxic activity and high feasibility for convenient “off‐the‐shelf” manufacturing. The manufacturing process for CAR‐NK cells is more convenient than for CAR‐T cells. Because there is no risk of GvHD, NK cells can be isolated from matched or HLA‐mismatched donors, offering more donor options and improving the quality of the final products. The low likelihood of GvHD and the diverse sources of NK cells may allow NK cells to be used as “off‐the‐shelf” products, readily available for clinical use [178]. Through engineering to express variety of receptors, cytokines, and suicide genes, CAR‐NK cells can be designed to target a variety of antigens, enhance in vivo proliferation and persistence, increase infiltration into solid tumors, and overcome challenges presented by resistant TME, ultimately leading to a more effective antitumor response [179, 180]. In mouse xenograft models of NSCLS, the transfection of anti‐B7‐H3 CAR into NK‐92MI cells resulted in a notable increase in the ability of NK‐92MI cells to destroy B7‐H3‐positive tumor cells. This heightened cytotoxicity was accompanied by a significant rise in the release of perforin/granzyme B and the expression of CD107a in anti‐B7‐H3 CAR‐NK‐92MI cells, indicating the potential use of B7‐H3‐specific CAR‐NK cells in adoptive cancer immunotherapy [181]. A study discovered that the gene mesenchymal‐epithelial transition factor (C‐Met) was increased in lung cancer tissues. The higher expression of c‐Met protein was linked to a worse prognosis for lung cancer patients. As a result, C‐Met‐CAR‐NK cells incorporating DAP10 as a beneficial activator for NK cell stimulation were created, it has been shown that this CAR cells by targeting C‐met is a promising strategy for the treatment of c‐Met‐positive cancers [182]. As previously stated, SCLC is distinguished by its elevated recurrence, resistance to drugs, and a restricted range of treatment options. Delta‐like ligand 3 (DLL3) has been identified as having heightened expression in SCLC, making it a plausible target for CAR NK cell immunotherapy. Studies have shown that coculturing DLL3^+^ SCLC cell lines with DLL3^‐^CAR NK‐92 cells resulted in substantial in vitro cytotoxicity and the generation of cytokines [183]. Despite the obvious advantages of CAR‐NK cells, the use of CAR‐NK cells encounters various hurdles. Initially, the process of increasing the number of NK cells in VITRO presents a challenge for CAR‐NK cell immunotherapy due to the inadequate quantity of NK cells obtained from a single donor for treatment. Furthermore, the effectiveness of CAR‐NK cells can be affected by the position of the CAR binding epitope and its closeness to the surface of the CAR‐NK cell. This has an impact on the ability of the CAR‐NK cell to bind to antigens and activate. Furthermore, the manufacturing procedure usually requires weeks to cultivate NK cells and produce cytokines. Obtaining NK cells is particularly problematic, as using one's own NK cells necessitates the process of freezing and thawing, which can reduce their effectiveness against tumors and their ability to survive. Moreover, the utilization of external cytokines may provide hazards such as systemic toxicity [184, 185, 186, 187].

A state‐of‐the‐art strategy in cancer immunotherapy involves targeting autophagy. A recent study demonstrated that rocaglamide (RocA), a natural compound, enhances the in vitro lysis of NSCLC cells by NK cells and induces tumor regression in vivo. RocA achieves this by suppressing autophagy, leading to the restoration of granzyme B levels from NK cells in NSCLC cells. This restoration increases the susceptibility of NSCLC cells to NK cell‐mediated killing. Additionally, RocA has been identified to target ULK1 (unc‐51 like autophagy activating kinase 1), a critical factor for initiating autophagy [188]. In an independent study, it was found that RocA caused damage to mitochondrial DNA (mtDNA) and promoted the release of mtDNA into the cytoplasm. This process activated the cGAS (cyclic GMP‐AMP synthase)‐STING (stimulator of interferon genes) signaling pathway, leading to increased infiltration of NK cells in NSCLC [189]. These findings position RocA as a potentially promising treatment option for individuals with NSCLC. The use of microRNA (miRNA) presents a potential treatment avenue for enhancing NK cell activity in NSCLC patients. It has been demonstrated that augmenting miR‐130a levels enhances the killing ability of NK cells against A549 cells. Further exploration reveals that miR‐130a targets STAT3, and overexpressing STAT3 negates the improved killing activity induced by miR‐130a in NK cells against NSCLC cells [190]. In the case of other rare respiratory system cancers, recent findings reveal an upregulation of lncRNA PVT1 in laryngeal cell lines. This increased expression of PVT1 influences laryngeal cell proliferation, apoptosis, and susceptibility to NK cells. Additionally, lncRNA PVT1 was found to enhance the expression of Muscleblind‐like proteins 1 (MBNL1), which are tissue‐specific regulators involved in RNA metabolism and pre‐messenger RNA splicing. This regulation impacts laryngeal cell progression by acting as a sponge for miR‐1301‐3p, a tumor suppressor, thereby presenting PVT1 as a novel target for NK cell‐related cancer immunotherapy [191]. A list of clinical trials utilizing NK cells to treat respiratory system cancers is presented in Table 2.

**Table 2:** List of ongoing/completed clinical trials using NK cell‐based approaches to treat respiratory system cancers.

Row	Study title	Phase	Trial no.	Interventions	Locations	Status
1	Clinical efficacy and safety of NK and NKT cells infusion in patients with nonsmall cell lung cancer	Phase 1	NCT03198923	Biological: NK cells and NKT cells	China	UNKNOWN
2	High‐activity natural killer immunotherapy for small metastases of nonsmall cell lung cancer	Phase 1/2	NCT03007875	Biological: high‐activity NK cells	China	COMPLETED
3	Natural killer cells and bortezomib to treat cancer	Phase 1	NCT00720785	Drug: Bortezomib Biological: NK cells	USA	COMPLETED
4	Clinical study on antitumor effect induced by activated primary natural killer (NK) Cells	Phase 1/2	NCT03634501	Biological: Activated NK cells	China	UNKNOWN
5	A clinical research of NK cell infusion combined with chemotherapy in the treatment of nonsmall cell lung cancer	Phase 2	NCT02734524	Biological: NK cells Drug: Taxol Drug: Carboplatin	China	UNKNOWN
7	Haploidentical NK cells after pemetrexed in patients with stage IV nonsmall cell lung cancer (NSCLC) (medi‐NK)	Phase 1	NCT03366064	Biological: Pemetrexed and donor‐derived NK cell infusion	South Korea	COMPLETED
8	A study to evaluate the safety and antitumor activity of SNK01 (NK cells) administered in combination with chemotherapy or chemotherapy/cetuximab in local advanced or metastatic nonsmall cell lung cancer patients who failed tyrosine kinase inhibitor treatment (SNK_ASTER)	Phase 1/2	NCT04872634	Biological: SNK01 (Super NK cell 01) Drug: GC Biological: Cetuximab	South Korea	UNKNOWN
9	Combined effect of natural killer cell and doublet chemotherapy in advanced NSCLC as the 1st line treatment (ANKL‐2)	Phase 2	NCT02370017	Biological: ANKL (Ex vivo‐expanded NK cell‐enriched lymphocytes)	South Korea	UNKNOWN
10	NK cell‐based immunotherapy as maintenance therapy for small‐cell lung cancer	Phase 2	NCT03410368	Biological: NK cells	China	UNKNOWN
11	Combination of cetuximab and NK immunotherapy for recurrent nonsmall cell lung cancer	Phase 1/2	NCT02845856	Drug: Cetuximab Biological: NK cell immunotherapy	China	COMPLETED
12	Genetically engineered natural killer (NK) cells with or without atezolizumab for the treatment of nonsmall cell lung cancer previously treated with PD‐1 and/or PD‐L1 immune checkpoint inhibitors	Phase 1	NCT05334329	Biological: Antineoplastic immune cell biological: Atezolizumab Procedure: Biospecimen collection Drug: Cyclophosphamide Drug: Fludarabine	USA	RECRUITING
13	Adoptive TKC transfer combined with chemotherapy for advanced nonsmall cell lung cancer (NSCLC)	Phase 1	NCT04990063	Drug: Chemotherapy Biological: Adoptive TKC transfer therapy	China	UNKNOWN
14	Combination of cryosurgery and NK immunotherapy for advanced nonsmall cell lung cancer	Phase 1/2	NCT02843815	Device: Cryosurgery Biological: NK cell Immunotherapy	China	COMPLETED
15	Donor natural killer cells, cyclophosphamide, and etoposide in treating children and young adults with relapsed or refractory solid tumors	Phase 1	NCT03420963	Biological: Cord Blood‐derived Expanded Allogeneic NK cells Drug: Cyclophosphamide Drug: Etoposide	USA	RECRUITING

## 8 Conclusion

**Figure 2:**
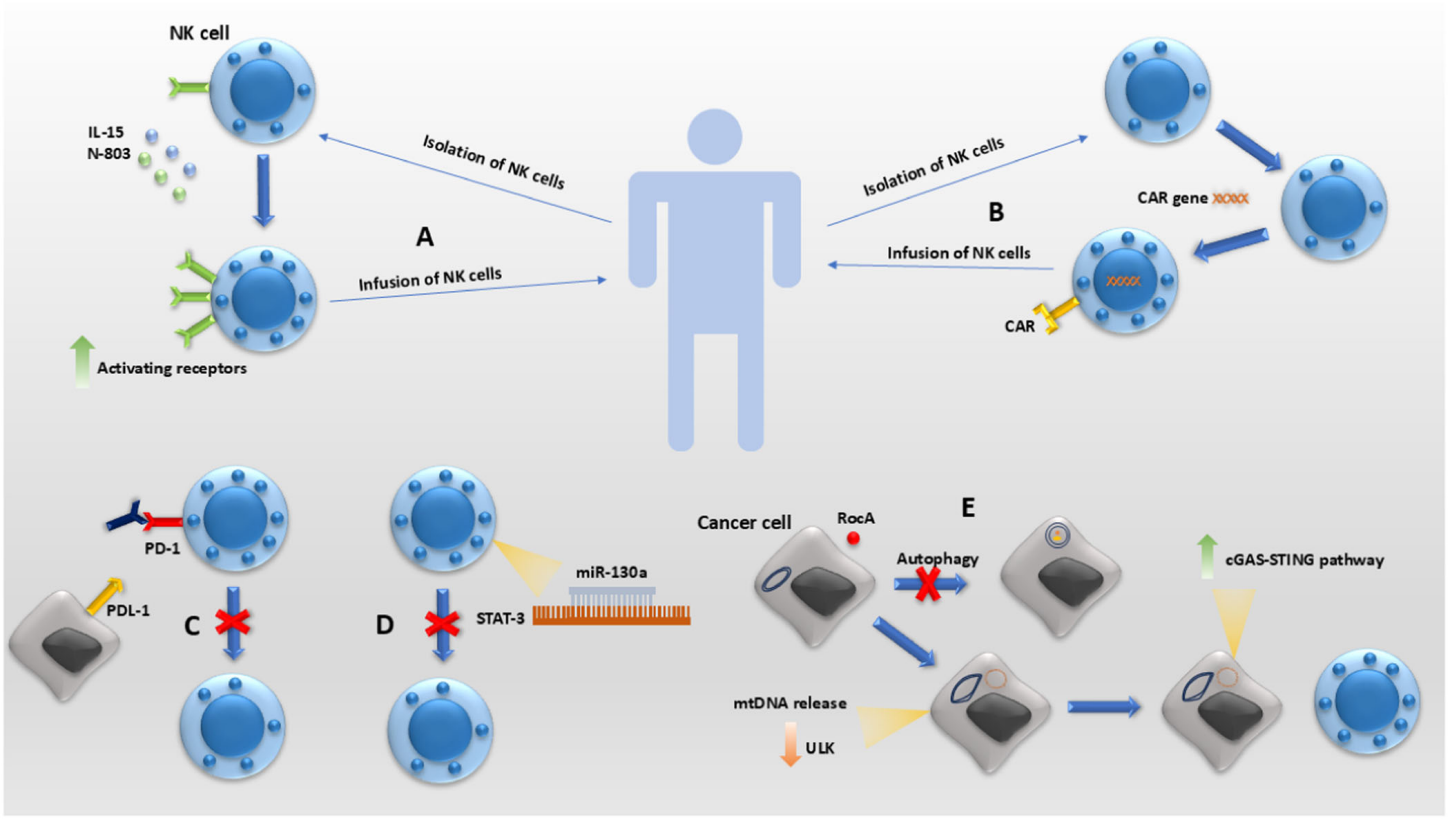
Various methods of NK cell‐based therapies used in fighting respiratory system cancers. (A) Isolated NK cells from the patient can be activated by cytokines and cytokine agonists and then infused to the patient's body or (B) can be genetically engendered by adding a genetic region coding for chimeric antigen receptor, which is able to recognize cancer cells. (C) Monoclonal antibodies can be used to attach to and block inhibitory receptors to avoid NK cell inhibition by inhibitory signals from cancer cells. (D) Introduction of miR‐130a into NK cells leads to the inhibition of STAT3, which is a negative regulator of NK cell function. (E) Rocaglamide (RocA) is used to inhibit autophagy in tumor cells by targeting ULK1, which results in mitochondrial damage and activation of the cGAS‐STING pathway. This enhances NK cell cytotoxic activity.

Cancers of the respiratory system are associated with significant morbidity and mortality, and their global burden continues to rise and affect people worldwide. NK cells play an important part in tumor surveillance by eliminating cancer cells that display diminished or absent MHC expression. However, the tumors also utilize several mechanisms to evade the vigilant guard of NK cells, which facilitates their proliferation and metastasis. This review explored the complex mechanisms of NK cell activation, involving various receptors such as NKG2D, CD16, and CD69, and their role in mediating processes such as ADCC in respiratory system cancers and also the challenges respiratory system tumors pose in evading NK cell‐mediated immune responses. Tumors employ various strategies, including physical barriers and altered chemokine environments, to hinder NK cell recruitment into the TME. Moreover, changes in vascularization, cytokine expression, and the upregulation of inhibitory factors such as PD‐1, PD‐L1, and TIGIT contribute to impairing NK cell function within the tumor. Of note, most studies were conducted on lung cancer and therefore, more attention should be paid on other cancers of respiratory system. Figure 2 shows various NK cell‐based immunotherapies used to treat respiratory cancers.

Despite the recent advances in cancer treatment, conventional therapies that rely on chemotherapy, radiation, and surgery pose significant challenges. Combination therapies involving NK cell‐based approaches, such as cytokine stimulation or immune checkpoint inhibitors, have shown promising results in clinical trials. The proven effectiveness of these approaches underscores the multifaceted potential of NK cells in addressing respiratory system cancers. The advent of CAR‐NK cells introduces a transformative facet, which offers improved safety profiles and adaptable engineering capabilities. Moreover, novel strategies targeting autophagy and utilizing miRNA modulation demonstrate the evolving landscape of NK cell‐based therapies. Despite challenges posed by factors such as the immunosuppressive microenvironment, overcoming these obstacles is key to unleashing the full potential of NK cells in reshaping the treatment landscape of respiratory system cancers. Additionally, further research is needed to identify novel targets for CAR‐NK cell therapy and explore combinatorial therapy options to maximize therapeutic efficacy. Moreover, the impact of etiologies of respiratory system cancers on the resulting tumors as targets for NK cell‐based therapies is another gap that should be addressed in the future studies.

## Author Contributions

**Maryam Dokhanchi:** conceptualization, investigation, writing–original draft, writing–review and editing. **Atefe Panahipoor Javaherdehi:** investigation, writing–original draft, writing–review and editing. **Mohammad Raad:** investigation, writing–original draft. **Shayan Khalilollah:** writing–original draft. **Pooya Mahdavi:** writing–original draft. **Mohammad Hossein Razizadeh:** conceptualization, data curation, investigation, supervision; visualization, writing–original draft, writing–review and editing. **Alireza Zafarani:** data curation, writing–original draft.

## Conflicts of Interest

The authors declare no conflicts of interest.

## Data Availability

All of the mentioned studies were cited in the paper.
